# Inhibiting tumor necrosis factor‐alpha at time of induced intervertebral disc injury limits long‐term pain and degeneration in a rat model

**DOI:** 10.1002/jsp2.1014

**Published:** 2018-05-25

**Authors:** Thomas W. Evashwick‐Rogler, Alon Lai, Hironobu Watanabe, Jonathan M. Salandra, Beth A. Winkelstein, Samuel K. Cho, Andrew C. Hecht, James C. Iatridis

**Affiliations:** ^1^ Leni and Peter W. May Department of Orthopaedics Icahn School of Medicine at Mount Sinai New York New York; ^2^ Keiyu Spine Center Keiyu Orthopedic Hospital Tatebayashi Japan; ^3^ Departments of Bioengineering and Neurosurgery University of Pennsylvania Philadelphia Pennsylvania

**Keywords:** axial back pain, discogenic pain, disc height, infliximab, intervertebral disc degeneration, low back pain, mechanical hyperalgesia, tumor necrosis factor‐alpha

## Abstract

**Background:**

Painful intervertebral disc (IVD) degeneration has tremendous societal costs and few effective therapies. Intradiscal tumor necrosis factor‐alpha (TNFα) is commonly associated with low back pain, but the direct relationship remains unclear.

**Purpose:**

Treatment strategies for low back pain require improved understanding of the complex relationships between pain, intradiscal pro‐inflammatory cytokines, and structural IVD degeneration. A rat *in vivo* lumbar IVD puncture model was used to 1) determine the role of TNFα in initiating painful IVD degeneration, and 2) identify statistical relationships between painful behavior, IVD degeneration, and intradiscal pro‐inflammatory cytokine expression.

**Methods:**

Lumbar IVDs were punctured anteriorly and injected with TNFα, anti‐TNFα, or saline and compared with sham and naive controls. Hindpaw mechanical hyperalgesia was assayed weekly to determine pain over time. 6‐weeks post‐surgery, animals were sacrificed, and IVD degeneration, IVD height, and intradiscal TNFα and interleukin‐1 beta (IL‐1β) expressions were assayed.

**Results:**

Intradiscal TNFα injection increased pain and IVD degeneration whereas anti‐TNFα alleviated pain to sham level. Multivariate step‐wise linear regression identified pain threshold was predicted by IVD degeneration and intradiscal TNFα expression. Pain threshold was also linearly associated with IVD height loss and IL‐1β.

**Discussion:**

The significant associations between IVD degeneration, height loss, inflammation, and painful behavior highlight the multifactorial nature of painful IVD degeneration and the challenges to diagnose and treat a specific underlying factor. We concluded that TNFα is an initiator of painful IVD degeneration and its early inhibition can mitigate pain and degeneration. Intradiscal TNFα inhibition following IVD injury may warrant investigation for its potential to alter downstream painful IVD degeneration processes.

## 1 INTRODUCTION

Low back pain has a lifetime prevalence of up to 85% and is the leading cause of disability worldwide.^1^, ^2^, ^3^ Intervertebral disc (IVD) degeneration is highly associated with back pain.^4^, ^5^, ^6^, ^7^, ^8^ However, many patients with magnetic resonance imaging evidence for IVD degeneration do not report back pain,^9^, ^10^ highlighting challenges in diagnosis and treatment. Consequently, nonsurgical interventions are most commonly recommended, and often of limited efficacy.^11^, ^12^ Surgery for disc degeneration has also yielded mixed clinical results. Specific phenotypes of painful IVD degeneration have been introduced to help distinguish aging from painful IVD degeneration conditions, and these phenotypes often involve structural defects and pro‐inflammatory conditions.^4^, ^7^, ^13^, ^14^, ^15^, ^16^ IVD injuries are known to precipitate a strong pro‐inflammatory responses with macrophage infiltration that can induce permanent alterations to IVD structure and function.^17^, ^18^, ^19^ Together, these findings demonstrate that IVD degeneration and pro‐inflammatory cytokines play important roles in back pain, but the relationships are complex and nonlinear. A better understanding of the relationship between IVD degeneration and back pain is an important societal research priority, and a clearer understanding may provide insights to identifying therapeutic targets and to developing preventative treatment strategies for discogenic pain.

IVD degeneration‐related pain is multifactorial and associated with intradiscal inflammation, neurovascular ingrowth into the IVD, sensitization of nervous system (ie, upregulation of pain‐related neuropeptides in the dorsal root ganglia), impingement of adjacent nerve roots, biomechanical instability, and increased demand on the surrounding spinal muscles.^20^, ^21^, ^22^, ^23^, ^24^ In particular, intradiscal pro‐inflammatory cytokines tumor necrosis factor‐alpha (TNFα) and interleukin‐1 beta (IL‐1β) are highly implicated in the development and progression of IVD degeneration and discogenic pain, and these cytokines can be produced by native nucleus pulposus (NP) and annulus fibrosus (AF) cells as well as infiltrating inflammatory cells.^18^, ^25^, ^26^, ^27^, ^28^, ^29^, ^30^, ^31^, ^32^, ^33^, ^34^ Upon IVD injury, macrophages and mast cells are recruited and release TNFα and IL‐1β in the IVD and also induce native IVD cells to further produce pro‐inflammatory cytokines, including TNFα, IL‐1β, IL‐6, and IL‐8.^35^, ^36^, ^37^ TNFα is highly associated with IVD degeneration because it has been demonstrated to promote extracellular matrix degradation via upregulation of catabolic mediators, including matrix metalloproteinases (MMPs) and a disintegrin and metalloproteinase with thrombospondin motifs (ADAMTS).^38^, ^39^, ^40^ TNFα can also upregulate substance P, nerve growth factor (NGF), and vascular endothelial growth factor production, which may induce painful conditions through sensitization of the nervous system and neurovascular ingrowth into the IVD.^41^, ^42^, ^43^, ^44^ The importance of TNFα in discogenic back pain led to multiple human clinical trials treating chronic discogenic pain with TNFα inhibitors, yet results were mixed highlighting a need for further research.^45^, ^46^, ^47^ There is a high research priority for investigating a direct relationship between TNFα and discogenic back pain that can further assess contribution of specific pain generators, including intradiscal pro‐inflammatory cytokines and structural IVD degeneration, and such a controlled investigation requires an animal study.

Animal models are commonly used for studying IVD degeneration, with pain often used as motivation for studying IVD degeneration. *In vivo* rodent models play an important role in determining the underlying pathophysiology of painful IVD degeneration because known quantitative methods are available that can characterize specific painful responses.^24^, ^48^ However, few studies have directly assessed the pain associated with IVD degeneration, and no study to date has investigated the direct associations between pain, structural degeneration, and intradiscal pro‐inflammatory cytokines in order to characterize the most important contributors to the painful process. Furthermore, rats offer advantages over mice because the larger size enables more precise control of induced injuries. Annular injury models (using puncture or stab injury) are commonly adopted to induce IVD degeneration in rat models because the severity of injury can be controlled. Our group previously developed an *in vivo* painful IVD degeneration model in rat and showed that AF puncture with intradiscal injection of phosphate buffered saline (PBS) produced painful behavior and degeneration in the injured IVDs, and intradiscal injection of TNFα into the injured IVD further increased painful behavior up to 6 weeks post‐injury.^48^, ^49^ The findings suggested that TNFα plays an important role in the development of IVD degeneration‐related pain; however, it is unclear whether inhibiting TNFα at the time of IVD injury can prevent or mitigate IVD degeneration and degeneration‐related pain. Furthermore, the relationships between pain, intradiscal pro‐inflammatory cytokines, and IVD structural degeneration in this model are unknown.

This study applied an *in vivo* rat model of painful IVD degeneration to determine (1) a role of TNFα in causing painful IVD degeneration following an IVD puncture injury; and (2) the relationships between pain, IVD structural degeneration, and intradiscal pro‐inflammatory cytokines in this model. We hypothesized that annular injury with intradiscal administration of TNFα would increase painful IVD degeneration, whereas blocking TNFα at the time of injury with intradiscal injection of anti‐TNFα would prevent this painful IVD degeneration cascade. We also hypothesized that the painful behavior would be multifactorial but be predicted by intradiscal pro‐inflammatory cytokines expression and IVD structural degeneration.

## 2 MATERIALS AND METHODS

### 2.1 Experimental design

A total of 36 skeletally mature^50^ male Sprague‐Dawley rats (4‐5 months old) were used and randomly assigned into one of five experimental groups: naive (*n* = 8), sham (*n* = 8), PBS (*n* = 8), TNFα (*n* = 6), or anti‐TNFα (*n* = 6). In the PBS, TNFα, and anti‐TNFα groups, rat lumbar IVDs were exposed, punctured, and intradiscally injected with PBS, TNFα, or anti‐TNFα, respectively. The injection of TNFα aimed to further increase the content of intradiscal TNFα in addition to the expected inflammatory response resulting from IVD injury; while anti‐TNFα was injected to downregulate the intradiscal TNFα response induced by IVD injury. The sham group involved surgery to expose lumbar IVDs only without puncture or injection, and naive group involved no surgical procedures at all. Pain behavior and IVD height were measured before surgery and then weekly after surgery using hindpaw mechanical hyperalgesia test and lateral radiographs, respectively. After pain behavior and radiographic measurements were taken at the 6‐week time point, the rats were euthanized, and the lumbar spines were isolated for morphological and biochemical analyses. All experimental procedures were approved by the Institutional Animal Care and Use Committee.

### 2.2 Lumbar IVD puncture and injection injury

**Figure 1:**
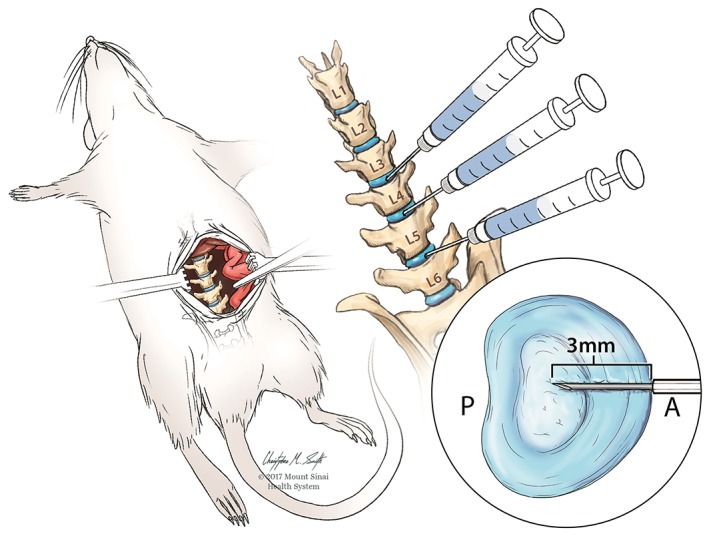
Induced intervertebral disc (IVD) injury illustration. Image created from Mount Sinai Health System. Used with permission

Surgery was performed under sterile conditions and general anesthesia (2% isoflurane in oxygen). The lumbar spine was accessed via an anterior approach with an abdominal midline incision. The lumbar spine was visualized, and the IVD levels were identified using the pelvic rim and aortic bifurcation as anatomical landmarks. L3/4, L4/5, and L5/6 IVDs were then punctured along the midline with a 26‐gauge needle at a depth of 3.0 mm guided by a needle stopper followed by intradiscal injection of PBS (2.5 μL), TNFα (0.25 ng in 2.5 μL; 80045RNAE50; Sino Biological Inc., Beijing, China),^49^ or anti‐TNFɑ (2.5 μL, 0.5 mg/kg; Remicade; Janssen Biotech Inc., Horsham, Pennsylvania),^51^ for PBS, TNFα, and anti‐TNFα groups, respectively, with all three IVDs receiving the same injectate (Figure 1). The anti‐TNFα agent, infliximab, is a monoclonal antibody that binds with high specificity to soluble and transmembrane TNFα and prevents TNFα from binding to its receptor thereby blocking its pro‐inflammatory effects. The peritoneum was then closed with 3‐0 silk sutures, and the skin was closed with 4‐0 nylon sutures. Rats were allowed *ad libitum* access to food and water. Rats were closely monitored for complications. Volume and method of intradiscal injection were determined from preliminary *in vitro* and *in vivo* studies demonstrating no visible fluid leakage or nerve irritation.

### 2.3 Pain behavior measurement

Severity of pain was measured using hindpaw mechanical hyperalgesia test, shown to be a sensitive measurement technique to quantify the pain associated with IVD degeneration in rats.^48^ A decrease in paw withdrawal threshold indicated an increase in pain sensitivity. The test was carried out in wire mesh‐floored cages, and the rats were placed in the cages at least 20 min prior to measurement for acclimation. Calibrated von Frey filaments, ranging from 0.4 to 26.0 g (Stoelting, Wood Dale, Illinois), were then applied to the plantar surface of the hindpaw with sufficient force to cause buckling of the filament. The 50% paw withdrawal thresholds were determined for both left and right hindpaws using the up‐down method with three trials for each side.^52^ The withdrawal thresholds from left and right hindpaws were then averaged for analysis and normalized to preoperative values.

### 2.4 Radiographic IVD height measurement

IVD heights of noninjured IVD (ie, L2/3 IVD) and injured IVDs (i.e. L4/5 and L5/6 IVDs) were measured via lateral radiographs with the animals under general anesthesia. Animals were anesthetized for approximately 10 min before taking the radiographs to minimize the effect of anesthesia on IVD height resulting from muscle relaxation and IVD swelling.^53^ After the radiographic film was digitized using a flatbed scanner with backlighting, the image was magnified and analyzed in ImageJ. Both upper and lower vertebral boundaries of the motion segment were manually identified, and coordinates of the vertebral boundaries were obtained. The coordinates were then transferred to the MATLAB (Mathworks, Inc., Natick, Massachusetts),^49^ which measured the averaged distance between the vertebral boundaries. A step wedge was used as a scale reference. Each disc was measured thrice and averaged for analysis. The IVD heights from the injured IVDs were also averaged for analysis and normalized by dividing by the presurgical heights of each IVD. Interrater and intrarater reliability tests were used to determine the consistency and repeatability for measuring IVD height using this technique. A total of 30 IVDs were randomly picked and measured by three researchers on two separate days with 7 days apart. The interrater reliability was excellent with intraclass correlation coefficient, ICC(3,3), of 0.983; and the interrater reliability was also excellent with ICC(3,3) for the three researchers of 0.992, 0.993, and 0.977 for the three researchers, respectively.

### 2.5 Tissue harvesting

Rats were euthanized at 6 weeks after surgery via carbon dioxide inhalation. Immediately after sacrifice, the lumbar spine (L1/2‐L3/4) was harvested, fixed in formalin, decalcified, embedded, and sectioned sagittally at 5 μm intervals.

### 2.6 IVD morphology and semi‐quantitative degeneration grading

Mid‐sagittal sections were stained with safranin‐O/light green/hematoxylin to assess IVD morphology and evaluated using bright‐field microscopy. The severity of IVD degeneration of each section was assessed using a semi‐quantitative IVD degeneration grading scale adapted from the grading scales proposed by Masuda et al^54^ and Rutges et al.^55^ The combined degeneration scale consisted of five categories, including (1) AF, (2) border between AF and NP, (3) cellularity of NP, (4) matrix of NP, and (5) endplate (Table 1). Each category received a score of 0‐2 with score 0 for normal morphology and score 2 for characteristic severe degeneration; therefore, the overall degenerative score for each section was between 0 and 10. All sections were assessed by two researchers blinded to the experimental groups. Intra‐ and inter‐rater reliability scores were obtained for error analysis purposes. Interrater reliability was excellent with intraclass correlation coefficient, ICC(1,3), for the two researchers of 0.961 and 0.968; and interrater reliability was excellent with intraclass correlation coefficient, ICC(1,3), of 0.887. Each section was graded twice at least 2 weeks apart by each researcher, and the four grades were then averaged for analysis.

**Table 1:** Intervertebral disc (IVD) degeneration grading scale^a^

Annulus fibrosus		
Grade	0	Normal, pattern of fibrocartilage lamellae (U‐shaped in the posterior aspect and slightly convex in the anterior aspect) without ruptured fibers and without a serpentine appearance anywhere within the annulus
	1	Ruptured or serpentine pattern fibers in less than 30% of the annulus
	2	Ruptured or serpentine pattern fibers in greater than 30% of the annulus
Border between annulus fibrosus and nucleus pulposus		
Grade	0	Normal
	1	Minimally interrupted
	2	Moderate/severe interruption
Cellularity of nucleus pulposus		
Grade	0	Normal cellularity with large vacuoles in the gelatinous structure of the matrix
	1	Slight decrease in the number of cells and fewer vacuoles
	2	Moderate/severe decrease (>50%) in the number of cells and no vacuoles
Matrix of nucleus pulposus		
Grade	0	Normal gelatinous appearance
	1	Slight condensation of the extracellular matrix
	2	Moderate/severe condensation of the extracellular matrix
Endplate		
Grade	0	Homogenous structure; regular thickness
	1	Slight irregularity with limited number of microfractures; locally decreased thickness
	2	Severe irregularity with multiple microfractures of endplate; generalized decrease of thickness

a The scale is a combination of previously developed and validated IVD degeneration grading schemes.^54^, ^55^

### 2.7 Intradiscal pro‐inflammatory cytokine expressions

Expression of intradiscal pro‐inflammatory cytokines TNFα and IL‐1β was determined using immunohistochemistry. Mid‐sagittal IVD sections were selected from each rat. Sections were rehydrated, treated with protein block reagent, and incubated overnight with either rabbit polyclonal primary antibody against rat TNFα (NB600‐587; Novus, Littleton, Colorado), rabbit polyclonal primary antibody against rat IL‐1β (bs‐6319R; Bioss, Woburn, Massachusetts), or normal rabbit serum (Biocare Medical, Concord, California) as negative control. Sections were incubated with a horseradish peroxidase‐conjugated anti‐rabbit secondary antibody (MP‐7451, ImmPRESS VR Reagent; Vector Laboratories, Burlingame, California) for 30 min and 3% hydrogen peroxide for 10 min, then treated with diaminobenzidine‐based horseradish‐peroxidase substrate (ImmPACT DAB; Vector Laboratories) to visualize the immunoreactivity. Sections were counterstained with toluidine blue, dehydrated, mounted, and evaluated using bright‐field microscopy.

Nine area‐of‐interest regions were identified from each IVD section, including outer anterior AF (three), inner anterior AF (two), NP (two), and posterior AF (two). The percentage of immunopositive cells from each area‐of‐interest region was determined via manual cell counting and then averaged for analysis.

### 2.8 Statistical analysis

The results of hindpaw mechanical hyperalgesia and IVD height obtained at different time points were normalized to those obtained before surgery to minimize interanimal variability, and the changes of the normalized results with time within and across experimental groups were analyzed using two‐way repeated measures ANOVA with Tukey's test as post hoc comparison. The morphological degeneration grades as well as percentages of intradiscal TNFα and IL‐1β immunopositive cells across experimental groups were compared using one‐way ANOVA with Tukey's test as post hoc comparison. The correlation between paw withdrawal threshold, IVD height, IVD degeneration grade, and percentage intradiscal TNFα and IL‐1β immunopositivities were analyzed using Pearson's correlation. A multivariate stepwise linear regression analysis was used to determine the predictive factors for paw withdrawal threshold, where all other outcome measures were defined as the independent variables. All the statistical analyses were conducted using Prism version 7 (GraphPad Software, Inc., La Jolla, California) and SPSS Statistics version 22 (IBM Corporation, Armonk, New York), and the level of significance was set to 0.05. D'Agostino and Pearson test demonstrated all data were normally distributed, so parametric tests were performed for data analyses.

## 3 RESULTS

### 3.1 Surgery did not affect rat general health

Both sham surgery and spinal injury procedures were well‐tolerated by the rats. The rat mean ±SD body weight of 495 ±48 g, 504 ±52 g, 522 ±51 g, 533 ±52 g, 544 ±53 g, 555 ±53 g, and 565 ±55 g, for presurgery and postsurgery weeks 1, 2, 3, 4, 5, and 6, respectively. There were no significant differences between groups at any time point. No intraoperative complications or obvious stress or discomfort were observed from the general physical examination.

### 3.2 TNFα inhibition at time of IVD injury prevents development of acute and long‐term painful behavior

**Figure 2:**
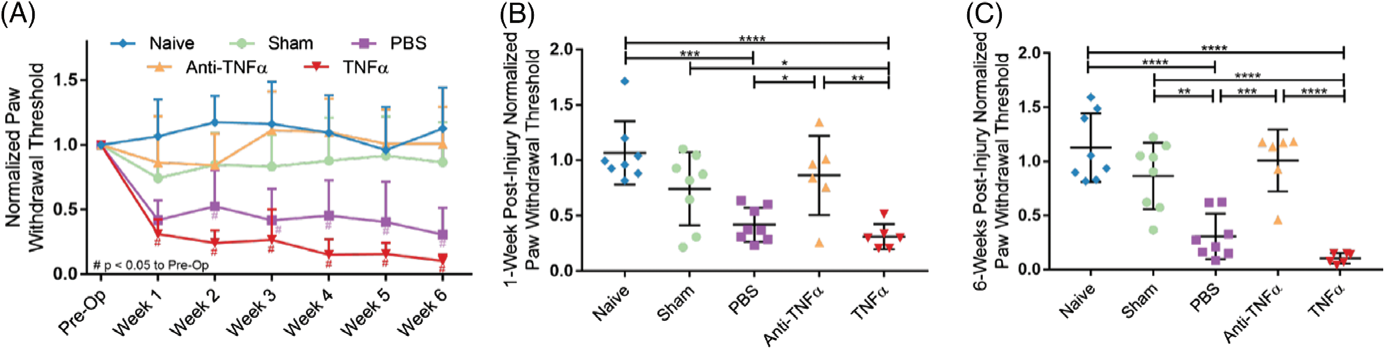
Tumor necrosis factor‐alpha (TNFα) inhibition at time of intervertebral disc (IVD) injury prevents development of acute and long‐term painful behavior. A, Normalized paw withdrawal threshold by experimental group over time. Scatter plots presents the comparison of normalized withdrawal threshold across groups at B (acute [1‐week postsurgery]) and C (long‐term [6 weeks postsurgery]) time points. Data presented as mean ± SD. # indicates *P* < .05 compared to presurgery baseline value. *P < .05, **P < .01, ***P < .001, ****P < .0001 by a one‐way ANOVA with Tukey's post hoc

The continuous changes of normalized hindpaw withdrawal threshold within each group, as well as the comparison of withdrawal threshold across groups at acute (ie, 1‐week postsurgery) and long‐term (ie, 6‐week postsurgery) were documented (Figure 2). Paw withdrawal thresholds were normalized to pre‐op levels for each animal. There were no significant changes in hindpaw withdrawal thresholds among naive and sham animals across the 6‐week duration of the study (Figure 2A). The paw withdrawal thresholds were significantly decreased following TNFα and PBS injections, and maintained throughout the duration of the 6‐week study (*P* < .05 at all time points compared to presurgery, Figure 2A). Their withdrawal thresholds were lower than both naive and sham groups for both 1‐week and 6‐week time points (*P* < .05, Figure 2B,C, respectively). However, intradiscal injection of anti‐TNFα prevented the decrease in paw withdrawal threshold induced by IVD injury and did not show significant change across the 6‐week duration of the study (*P* > .05 compared to presurgery for all time points). Paw withdrawal thresholds following anti‐TNFα injection were significantly higher than following both PBS and TNFα injections at both 1‐week and 6‐week time points (*P* < .05 with both groups at both time points).

### 3.3 TNFα inhibition at time of IVD injury prevents IVD structural degeneration but not height loss

**Figure 3:**
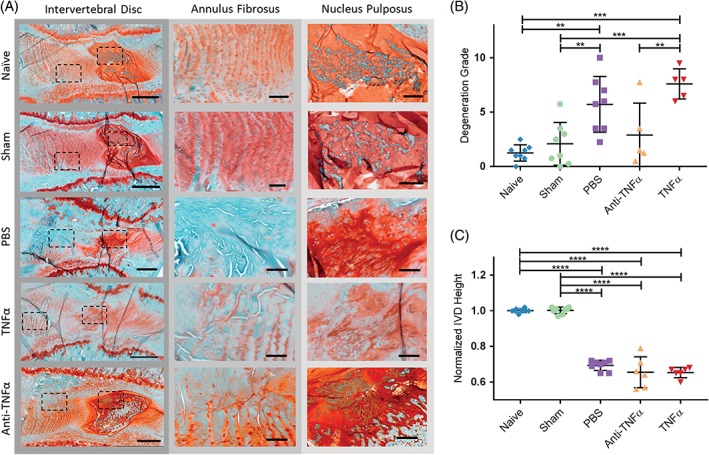
Tumor necrosis factor‐alpha (TNFα) inhibition at time of intervertebral disc (IVD) injury prevents IVD structural degeneration but not IVD height loss. A, Mid‐sagittal sections stained with hematoxylin/safranin‐O/light green. Boxed regions shown under higher magnification. B, IVD degeneration by group. C, Radiographic IVD height by group normalized to presurgery levels. Scale bars (whole IVD) = 500 μm. Scale bars (annulus fibrosus [AF] and nucleus pulposus [NP]) = 100 μm. Data presented as mean ± SD. *P < .05, **P < .01, ***P < .001, ****P < .0001 by a one‐way ANOVA with Tukey's post hoc

Normal IVD morphology was observed in both naive and sham animals. AF puncture with intradiscal injections of PBS, TNFα, or anti‐TNFα induced observable degenerative changes, including smaller and more fibrous NP, decreased number of NP cells, less distinct NP‐AF boundary, and disorganized AF lamellae (Figure 3A). There was no evidence of NP herniation in any IVD upon histological analysis.

The semi‐quantitative degeneration grading scale showed that both naive and sham IVDs had minimal degeneration with mean ±SD degeneration grade of 1.25 ±0.74 and 2.09 ±1.97, respectively (Figure 3B). PBS‐injected (5.72 ±2.57) and TNFα‐injected (7.60 ±1.39) IVDs had significantly increased degeneration than both naive and sham IVDs and some evidence of increased glycosaminoglycan loss. Anti‐TNFα‐injected (2.90 ±2.93) IVDs had significantly lower degeneration than TNFα‐injected IVDs but did not show significant difference from naive‐, sham‐, or PBS‐injected IVDs (*P* = .615. *P* = .955, and *P* = .133, respectively).

The IVD heights obtained at 6‐week postsurgery were normalized to presurgery baseline. The normalized heights of all injured IVDs (including PBS, TNFα, and anti‐TNFα) were significantly smaller than those of naive and sham IVDs (Figure 3B). There was no significant difference between naive and sham groups, or among the three injury groups (Figure 3C).

### 3.4 TNFα inhibition at time of IVD injury prevents long‐term upregulation of intradiscal TNFα

**Figure 4:**
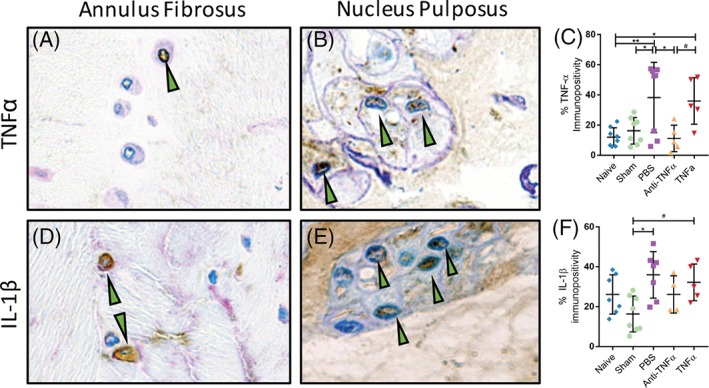
Tumor necrosis factor‐alpha (TNFα) inhibition at time of intervertebral disc (IVD) injury prevents intradiscal TNFα upregulation at 6 weeks postsurgery. Representative anti‐TNFα immunostained sections for A (annulus fibrosus) and B (nucleus pulposus). C, Quantified TNFα by group. Representative anti‐interleukin (IL)‐1β immunostained sections for D (annulus fibrosus) and E (nucleus pulposus). F, Quantified IL‐1β by group. Green arrows denote immunopositive cells. Scatter plots presents the percentage of immunopositive cells for E (TNFα) and F (IL‐1β). Data presented as mean ± SD. *P < .05, **P < .01, #*P* < .10 by a one‐way ANOVA with Tukey's post hoc

At 6‐week postsurgery, compared to naive and sham IVDs, the percentage of intradiscal TNFα immunopositive cells was increased following PBS or TNFα injections. However, intradiscal injection of anti‐TNFα alleviated the changes in intradiscal TNFα immunopositivity induced by IVD injury and showed no significant difference from both naive or sham IVDs (Figure 4E).

In contrast, the percentage of intradiscal IL‐1β immunopositive cells was only increased following PBS injection relative to sham at 6‐week postsurgery. IL‐1β trends to increase in TNFα‐injected IVDs relative to sham. The anti‐TNFα‐injected IVDs did not show significant difference in intradiscal IL‐1β immunopositivity from both naive or sham IVDs (Figure 4F).

### 3.5 Painful behavior predicted by IVD degeneration using stepwise linear regression analysis

**Figure 5:**
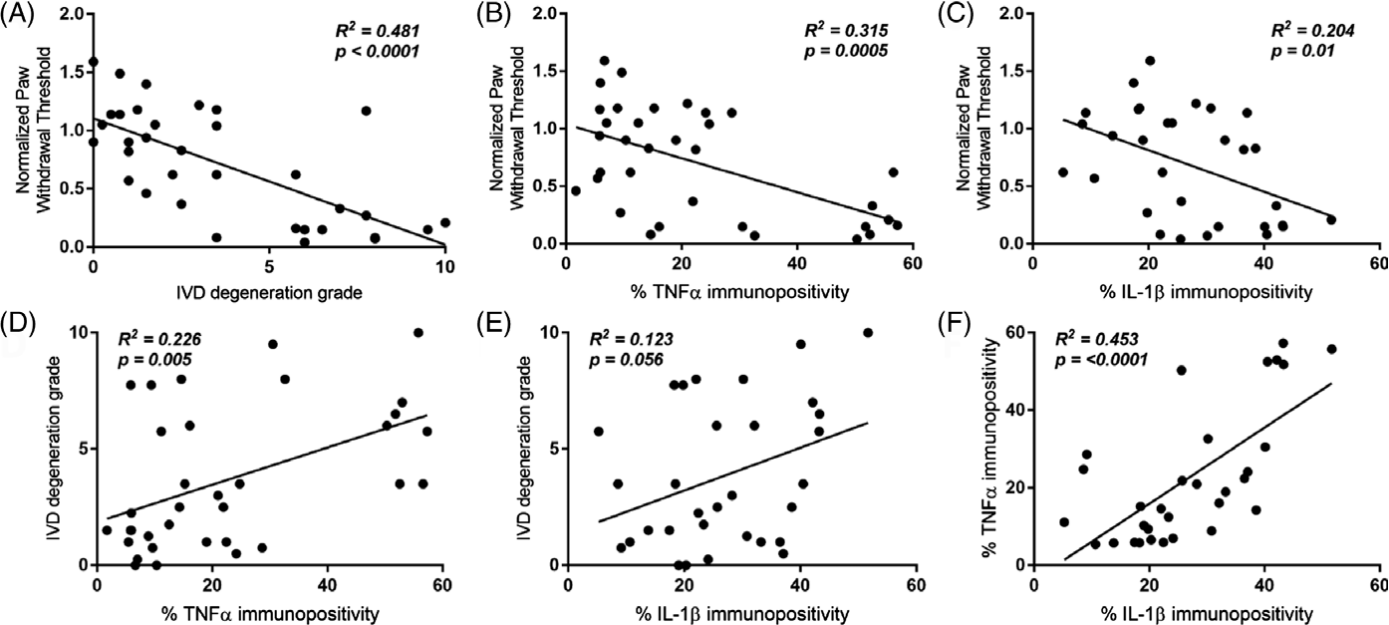
Pain, structural parameters, and intradiscal pro‐inflammatory cytokines correlate. A, Normalized paw withdrawal threshold vs intervertebral disc (IVD) degeneration. B, Normalized paw withdrawal threshold vs intradiscal tumor necrosis factor‐alpha (TNFα) immunopositivity. C, Normalized paw withdrawal threshold vs intradiscal interleukin (IL)‐1β immunopositivity. D, IVD degeneration vs intradiscal TNFα immunopositivity. E, IVD degeneration vs intradiscal IL‐1β immunopositivity. F, Intradiscal TNFα immunopositivity vs intradiscal IL‐1β immunopositivity

Multivariate stepwise linear regression analysis showed that the normalized paw withdrawal threshold could be significantly predicted by IVD degeneration grade (*β* = −0.533, *P* = .001) and intradiscal TNFα immunopositivity (*β* = −0.355, *P* = .016) (Table 2). Normalized paw withdrawal threshold was also linearly correlated to IVD degeneration grade and intradiscal TNFα and IL‐1β immunopositivity with *R* ^2^ of 0.481, 0.315, and 0.204, respectively (Figure 5A‐C). IVD degeneration grade correlated with intradiscal TNFα and IL‐1β immunopositivity with *R* ^2^ of 0.226 and 0.123, respectively (Figure 5D,E). Intradiscal TNFα and IL‐1β immunopositivity with *R* ^2^ of 0.453 (Figure 5F).

**Table 2:** Painful behavior predicted by intervertebral disc (IVD) degeneration and intradiscal tumor necrosis factor‐alpha (TNFα)^a^

	Multivariate	
	*β*	*P*‐value
IVD degeneration	**−0.533**	**0.001**
Intradiscal % TNFα positivity	**−0.355**	**0.016**
Intradiscal % interleukin‐1 beta positivity	−0.048	0.777
Normalized IVD height	−0.026	0.876

*β*, standardized coefficient of variation. Bold indicates statistical significance.

a Relationship of paw withdrawal threshold to structural IVD degeneration, IVD height, and intradiscal pro‐inflammatory cytokines using multivariate stepwise linear regression.

### 3.6 Pain threshold, structural degeneration and intradiscal pro‐inflammatory cytokines correlate with each other

Pearson's correlation analysis showed that the normalized paw withdrawal threshold, IVD degeneration grade, normalized IVD height, as well as intradiscal TNFα and IL‐1β immunopositivity were significantly correlated with each other (*P* < .05), except for the association between IVD degeneration and intradiscal IL‐1β expression, which showed a trend of correlation with *R* ^2^ of 0.351 (*P* = .053) (Table 3).

**Table 3:** Pain, structural intervertebral disc (IVD) degeneration, and intradiscal pro‐inflammatory cytokines correlate

		IVD degeneration	Normalized IVD height	% Intradiscal TNFα positivity	% IntradiscalI L‐1β positivity
Normalized paw withdrawal threshold	*r* (*P*)	**−0.694 (<0.001)**	**0.508 (0.002)**	**−0.562 (0.001)**	**−0.452 (0.011)**
IVD degeneration	*r* (*P*)	1	**−0.630 (<0.001)**	**0.476 (0.004)**	**0.351 (0.05)**
NormalizedIVD height	*r* (*P*)		1	**−0.407 (0.017)**	**−0.466 (0.008)**
% Intradiscal TNFα positivity	*r* (*P*)			1	**0.674 (<0.001)**

Abbreviations: IL‐1β, interleukin‐1 beta; TNFα, tumor necrosis factor‐alpha. Bold indicates significant correlations.

## 4 DISCUSSION

This study identified multifactorial causes of painful IVD degeneration and determined a causal role for TNFα on the initiation of painful IVD degeneration in an *in vivo* rat IVD puncture and injection model. Intradiscal TNFα was directly and causally involved in the initiation of a painful IVD degeneration cascade because anti‐TNFα injection into lumbar IVDs at the time of IVD puncture injury reduced pain and IVD degeneration grade to sham levels. IVD degeneration grade and intradiscal TNFα expression were identified as predictive factors for IVD degeneration‐related pain using stepwise multivariate regression, while univariate correlation analysis further identified associations of pain with IVD height loss and intradiscal IL‐1β expression.

This study injected anti‐TNFα intradiscally at the time of injury and identified TNFα as an essential initiator of a painful IVD degeneration cascade. Relatively few animal models exist that identify causative factors underlying painful IVD degeneration without direct herniation. Annular puncture with intradiscal injection of TNFα previously demonstrated significantly increased IVD degeneration and increased painful behavior, similar to the current study which showed enhanced pain sensitivity behavior with either TNFα or PBS injection.^49^ Intradiscal injection of complete Freund's adjuvant into the rat lumbar IVD also significantly increased painful behavior in rats *in vivo* as measured by the hindpaw mechanical hyperalgesia test.^56^ Both of these studies implicate TNFα and intradiscal pro‐inflammatory cytokines more generally as sufficient to initiate a painful IVD degeneration cascade, yet neither determined if TNFα was an essential initiator of the painful IVD degeneration. Taken together with the literature, the results from this study suggest local TNFα inhibition at early stages after IVD injury may offer potential benefit for preventing painful IVD degeneration progression, although immediate intradiscal injection of anti‐TNFα would be difficult to directly translate to the human clinical condition.

The moderate to severe IVD degeneration of the current study is comparable to other injury models in the literature. Previous puncture injury studies demonstrated that IVD degeneration occurs with needle puncture, and IVD degeneration severity increased with needle diameter and needle puncture depth.^49^, ^54^, ^57^, ^58^, ^59^ Needle puncture injuries with complete AF puncture (ie, into the NP) with larger needle diameters (~50% IVD height) induced moderate to severe IVD degeneration, similar to the current study. Multiple studies also inject pro‐inflammatory factors or matrix degrading enzymes to induce matrix changes more representative of chronic degeneration conditions in acute puncture injury models. Specifically, annular puncture with chondroitinase ABC, complete Freund's adjuvant, and TNFα have all been used to induce moderate to severe IVD degeneration with significantly reduced IVD height, diminished proteoglycan content, loss of NP cells, and altered IVD biomechanics.^49^, ^56^, ^60^, ^61^ While these studies contextualize the current model of painful IVD degeneration as a model of moderate to severe IVD degeneration, several comprehensive reviews of other animal models of IVD degeneration exist.^24^, ^62^, ^63^, ^64^

TNFα inhibition has been studied clinically for treating low back pain; however, the effects are inconclusive. Patient‐reported levels of pain and disability were decreased up to 8 weeks after intradiscal administration of the TNFα inhibitor etanercept (2 mL bupivacaine with 10 mg etanercept) in a prospective, randomized clinical trial.^45^ Similar findings were observed in a retrospective analysis, which showed perispinal delivery of etanercept (25 mg) significantly reduced pain and weakness in patients with severe, chronic discogenic pain.^46^ However, another study found no reduction in patient‐reported pain 1‐month after intradiscal etanercept administration in a double‐blind placebo‐controlled study.^47^ The current findings in the context of the more mixed human clinical literature suggest that TNFα inhibition immediately after acute, traumatic IVD injury, or possibly early in the IVD degenerative process offers promise for mitigating a painful degenerative cascade. We speculate the limited effectiveness of TNFα inhibition in clinical studies may be the result of administration long after the onset of discogenic pain, so the timing of anti‐TNFα administration is of utmost importance. Future studies using the current animal model are warranted to determine the importance of TNFα inhibition timing, specifically whether intradiscal anti‐TNFα injection can reduce pain after the onset of painful IVD degeneration. However, we also provided local treatment at the source of pain in this model and it is possible that some of the limited efficacy of the clinical trials is due to ineffective dosing at the source of the pain. Further investigations are also warranted to assess dosing effects and administration routes, and to investigate TNFα inhibition in chronic painful IVD degeneration conditions clinically.

In this *in vivo* rat study, the pain behavior was significantly associated with IVD degeneration and intradiscal TNFα expression as predictive factors using multivariate stepwise linear regression, and univariate correlation analysis also showed that the pain was significantly associated with loss of IVD height and intradiscal IL‐1β expression. These results are consistent with another study that used an annular puncture model with NP removal that found a correlation between IVD degeneration and mechanical hyperalgesia, which was assayed directly on the low back.^65^ The multiple associations of pain with IVD structural and biochemical degenerative changes in this *in vivo* rat model have many similarities with painful IVD degeneration in humans, which supports the feasibility of using this rat model to understand the pathophysiology of discogenic pain.^4^, ^6^, ^13^, ^66^, ^67^, ^68^ The lack of significant herniation or endplate damage in the observed IVD degeneration in this study is suggestive of intradiscal pro‐inflammatory cytokines as a cause for pain. It should be noted that anti‐TNFα prevented IVD degeneration but not a loss of IVD height. We believe this is because anti‐TNFα mitigated some of the inflammation‐induced matrix catabolism, as observed by decreased IVD degeneration, but did not restore the IVD height loss caused by the puncture.

We chose to study the role of TNFα in initiating discogenic pain and the effect of its inhibition because it is thought to initiate an inflammatory cascade, which is then propagated by other pro‐inflammatory cytokines. TNFα stimulates IL‐1β expression, which plays a significant role in upregulating catabolic MMPs and ADAMTS enzymes.^38^, ^69^, ^70^, ^71^ Bovine IVDs cultured in the presence of TNFα had increased gene expression of multiple pro‐inflammatory and catabolic factors after 7 days, which persisted to 21 days without recovery after TNFα was removed from culture.^70^ Similarly, human NP cells cultured with TNFα increased gene expression of IL‐1β, IL‐6, and IL‐8, which was inhibited by early anti‐TNFα administration.^72^ Interestingly, human NP cells cultured with TNFα significantly upregulated IL‐1β gene expression, and NP cells treated with IL‐1β significantly upregulated MMPs to a much greater extent than when treated with TNFα,^69^ further highlighting the role of TNFα in inflammation initiation and of IL‐1β in enhancing catabolism. Accordingly, we believed inhibiting specifically TNFα at the time of annular injury would have the greatest effect on the inflammatory response, which the current data support. This study adds to the literature by showing that TNFα plays a key role in initiating a painful inflammatory cascade, which early inhibition can prevent. A previous clinical study found the serum half‐life of infliximab, the TNFα inhibitor used in this study, is 7 to 12 days when given intravenously.^73^ While the IVD is avascular and infliximab may have remained in the IVD for a longer period of time, it is highly unlikely that the original infliximab remained present at the 6‐week end point. Thus, the significant decrease in intradiscal TNFα after intradiscal anti‐TNFα injection suggested that intradiscal anti‐TNFα administration was effective in inhibiting the inflammatory cascade in response to IVD puncture injury.

Furthermore, TNFα and intradiscal pro‐inflammatory cytokines more generally are known to be associated with multiple pain pathways.^16^, ^27^, ^33^, ^34^, ^74^, ^75^ TNFα can induce inflammatory pain as it directly sensitizes sensory neurons through a reduction in the firing thresholds, which contributes to maintenance of pain.^76^, ^77^, ^78^, ^79^, ^80^, ^81^ An electrophysiology study demonstrated TNFα application to rat nociceptive neurons decreased response latency and increased spontaneous firing frequency, supporting the role of TNFα sensitizing peripheral nerves on the cellular level.^76^ TNFα has also been shown to induce pain on the behavioral level. TNFα injection directly into rat hindpaws resulted in increased hyperalgesia demonstrating a role of TNFα in the initiation of inflammatory pain, and this painful behavior was inhibited by local TNFα inhibition, demonstrating the potential of local TNFα inhibition to alleviate the inflammatory pain in the periphery.^82^ In an investigation of the inflammatory response and effect of anti‐TNFα on pain in a surgically induced IVD herniation model with autologous IVD autograft and spinal nerve ligation, local application of anti‐TNFα at the time of herniation surgery significantly improved paw withdrawal threshold in a rat model.^37^ These finding further support the role of TNFα in peripheral inflammatory pain and the concept of locally inhibiting TNFα at early stages after injury to prevent the development of long‐term pain. However, it should be noted that the pain pathophysiology is different between the two models. The pain pathophysiology is much clearer in IVD herniation models with inflamed IVD tissue directly impinging a nerve but more difficult to identify in discogenic pain models, which highlights the importance of the validation in the current model. TNFα upregulates NGF in IVD cells, and nociceptive nerves, usually restricted to the outer AF in normal healthy IVDs, can be stimulated with NGF to grow into the inner AF and NP as was found in the painful human IVDs.^23^, ^41^, ^83^, ^84^ In this model, anti‐TNFα may have worked in several different ways: mitigating intradiscal inflammation, thereby preventing innervated nerve stimulation, decreasing the inflammatory environment of adjacent nerve roots, or inhibiting neovascular innervation. Likely, the mitigation of pain behavior following annular injury is a combination of the aforementioned factors, and the specific mechanism requires future investigations. However, histological assessments with this model did not identify obvious nerves or vessels, suggesting neovascularization is not likely to be a dominant cause of observed pain.

The current study focused on the changes in painful behavior and the structural and biochemical changes in the IVD in response to IVD injury and was limited because we did not investigate changes in the central nervous system, which plays important roles in both acute and chronic pain and warrants future investigation. Previous work has shown TNFα upregulates NGF in NP and AF cells in vitro,^41^ so annular puncture with intradiscal injections of TNFα or PBS may facilitate nerve ingrowth and upregulation of neurotropic factors including NGF and VEGF, while anti‐TNFα injection mitigates nerve ingrowth and expression of these neurotropic factors. Similarly, local anti‐NGF treatment attenuated the development of mechanical hyperalgesia in a rat facet joint distraction model,^85^ so there is some evidence to support TNFα and NGF play important roles on pain initiation. Understanding the relationship of other inflammation‐related proteins and NGF to pain and the broader inflammatory changes after TNFα injection or inhibition is an interesting area of future investigation. Additionally, only male rats were used in this study to minimize variability in behavioral and biochemical measurements, and it should be noted that there is evidence that pain pathways differ between sexes.^86^, ^87^ While it is not expected that the conclusions of the current findings would differ across sexes, male and female rats likely have sex differences in the neural pathophysiology of painful IVD degeneration that warrant further investigation. Lastly, rodent IVDs contain different cell types to those during human degeneration, and thus the responses may vary. Nonetheless, rodents are widely used to study IVD pathologies and treatment strategies.^88^

In conclusion, the results demonstrate that intradiscal TNFα plays a critical role initiating an inflammatory cascade that results in painful IVD degeneration and suggest that inhibiting TNFα at early stages after IVD injury may offer potential benefit for preventing pain symptoms and structural IVD degeneration. The pain behavior following IVD injury was significantly associated with IVD degeneration and intradiscal pro‐inflammatory cytokine expressions, which supports that this *in vivo* rat model has a phenotype similar to the human condition of painful IVD degeneration and could be a useful tool to study the underlying pathophysiology of painful IVD degeneration and for screening therapeutic strategies for discogenic pain.
